# Markers and makers of NKT17 cells

**DOI:** 10.1038/s12276-023-01015-y

**Published:** 2023-06-01

**Authors:** Nurcin Liman, Jung-Hyun Park

**Affiliations:** Experimental Immunology Branch, Center for Cancer Research, National Cancer Institute, NIH, Bethesda, MD 20892 USA

**Keywords:** NKT cells, Innate immunity, Lymphocyte differentiation

## Abstract

Invariant natural killer T (*i*NKT) cells are thymus-generated innate-like αβ T cells that undergo terminal differentiation in the thymus. Such a developmental pathway differs from that of conventional αβ T cells, which are generated in the thymus but complete their functional maturation in peripheral tissues. Multiple subsets of *i*NKT cells have been described, among which IL-17-producing *i*NKT cells are commonly referred to as NKT17 cells. IL-17 is considered a proinflammatory cytokine that can play both protective and pathogenic roles and has been implicated as a key regulatory factor in many disease settings. Akin to other *i*NKT subsets, NKT17 cells acquire their effector function during thymic development. However, the cellular mechanisms that drive NKT17 subset specification, and how *i*NKT cells in general acquire their effector function prior to antigen encounter, remain largely unknown. Considering that all *i*NKT cells express the canonical Vα14-Jα18 TCRα chain and all *i*NKT subsets display the same ligand specificity, i.e., glycolipid antigens in the context of the nonclassical MHC-I molecule CD1d, the conundrum is explaining how thymic NKT17 cell specification is determined. Mapping of the molecular circuitry of NKT17 cell differentiation, combined with the discovery of markers that identify NKT17 cells, has provided new insights into the developmental pathway of NKT17 cells. The current review aims to highlight recent advances in our understanding of thymic NKT17 cell development and to place these findings in the larger context of *i*NKT subset specification and differentiation.

## Immunology: Investigating a subset of natural killer T cells

A subset of white blood cells called invariant natural killer T (*i*NKT) cells secrete the cell-signaling molecule interleukin 17 (IL-17) which promotes immune reactions that can be either protective or damaging to the organism. *i*NKT cells can eliminate diseased cells, including cancer cells or pathogen-infected cells. NKT17 cells are the IL-17-producing subset of *i*NKT cells. Nurcin Liman and Jung-Hyun Park at the US National Cancer Institute, Bethesda, review recent advances in understanding how NKT17 cells undergo development in the thymus to acquire differing functions and phenotypes. The authors consider insights and distinct hypotheses focused on the key question of how and when the development of NKT17 cells diverges from that of other subsets of *i*NKT cells, which has implications in understanding their effector functions and roles among different tissues.

## Introduction

The antigen receptor of T cells, commonly referred to as the T cell receptor (TCR), is a defining feature of all T cells. The TCR is not only required for the generation of T cells in the thymus but also determines their antigen specificity, lineage choice, phenotype, and function, among other characteristics^1^. Consequently, the forced expression of a pre-rearranged TCR was found to determine the CD4 versus CD8 lineage fate as well as to control the choice of helper versus cytotoxic function and other characteristics of developing thymocytes^2–4^. Because the random, somatic recombination of TCR genes permits the generation of a vastly diverse TCR repertoire, conventional αβ T cells have access to a large pool of different TCR specificities to choose from for their TCR expression during thymic development^5,6^. In agreement, the TCRs of mature T cells comprise a highly diverse antigen repertoire^7,8^. Curiously, some T cells abstain from making use of the diverse TCR repertoire and instead employ a highly limited set of TCR chains for their generation. Because the cellular identity of T cells is imposed by the TCR, such oligoclonal T cells share common features in their antigen specificity and phenotype, and they can be pooled into specific T cell subsets with distinct features and functions.

Invariant natural killer T (*i*NKT) cells are prime examples of such an oligoclonal T cell population, and they are distinct from conventional αβ T cells in several aspects related to their phenotype and effector functions^9,10^. Foremost, *i*NKT cells are heavily constrained in their TCR repertoire, as they all express the canonical Vα14-Jα18 TCRα chain in association with a TCRβ chain that is limited in its diversity, being Vβ2, Vβ7, or Vβ8^11,12^. Importantly, the invariant Vα14-Jα18 TCRα chain restricts TCR binding to nonclassical MHC molecules, specifically to the nonclassical MHC-I-like molecule CD1d, that are bound to glycolipids^13,14^. The glycosphingolipid α-galactosylceramide (α-GalCer) is the prototypic antigen recognized by *i*NKT cells^13,15^, but other glycolipid analogs also have been identified to bind CD1d and be capable of activating *i*NKT cells^14,16^. Structural analyses revealed that the spatial interaction between glycolipid-loaded CD1d and the *i*NKT TCR is asymmetric and depends heavily on the invariant TCRα chain, with minimal contributions from the TCRβ chain, to form the binding interface^17,18^. Consequently, *i*NKT cells, which by definition all express the canonical Vα14-Jα18 TCRα chain, display the same MHC restriction and respond to the same antigens. As a corollary, all *i*NKT cells react to glycolipid-bound CD1d molecules and therefore can be identified by α-GalCer-loaded recombinant CD1d tetramers^19–21^. Thus, *i*NKT cells correspond to a subset of αβ T cells that are highly constrained in their TCR repertoire and thus share a limited antigen specificity.

Given that all *i*NKT cells express the same invariant TCRα chain and react to the same antigen, it could be assumed that the phenotypes and effector functions of individual *i*NKT cells would be uniform. Contrary to this expectation, the *i*NKT cell pool comprises many different subsets that are highly heterogeneous in their effector function, coreceptor expression, and tissue distribution^22,23^. Long-standing efforts to stratify these different *i*NKT cells have led to the proposal of different subsets of *i*NKT cells, either based on CD4 coreceptor expression^24,25^, developmental stage^26^, or effector function^27,28^. In the last case, *i*NKT cells could be classified into distinct subsets based on their cytokine production. Analogous to the Th1, Th2, and Th17 CD4 T helper subsets, *i*NKT cells that primarily express IFNγ, IL-4, or IL-17 have been referred to as NKT1, NKT2, and NKT17 cells, respectively^27^. These cytokine-based *i*NKT subsets can also be identified by their transcription factor expression profiles, whereby T-bet is exclusively expressed in NKT1 cells and RORγt is expressed in only NKT17 cells^27,28^. Similar to Th2 cells, NKT2 cells express high levels of GATA-3, but GATA-3 expression is not exclusive to NKT2 cells^29^. Thus, high-level expression of another transcription factor, the promyelocytic leukemia zinc-finger (PLZF) protein, is frequently used for more stringent identification of NKT2 cells^27,30–33^. Overall, *i*NKT cells comprise different functional subsets that display the same TCR specificity. As such, the holy grail for the understanding of *i*NKT subset specification is the elucidation of the mechanism through which the same TCR specificity can give rise to at least three distinct functional subsets; this is a highly active area of research^10,23,34^.

*i*NKT cells comprise only a small population of T cells, but they play disproportionally important roles in immune regulation and surveillance^35–37^. Specifically, IL-17-producing NKT17 cells have been implicated in both host defense against fungal infections and the pathogenesis of many autoimmune diseases, such as asthma and psoriasis, as well as graft-versus-host disease^38–40^. Nonetheless, how and when the developmental pathway of NKT17 cells diverts from those of other *i*NKT subsets remain mostly unknown, and why the frequency and number of NKT17 cells vary among different tissues and mouse strains is also unclear^39,41–43^. Here, we review and discuss recent progress in the field that has addressed these questions and provide a summary of different markers that identify NKT17 cells and how their expression is associated with NKT17 cell development and differentiation.

### *i*NKT cell development in the thymus

**Fig. 1:**
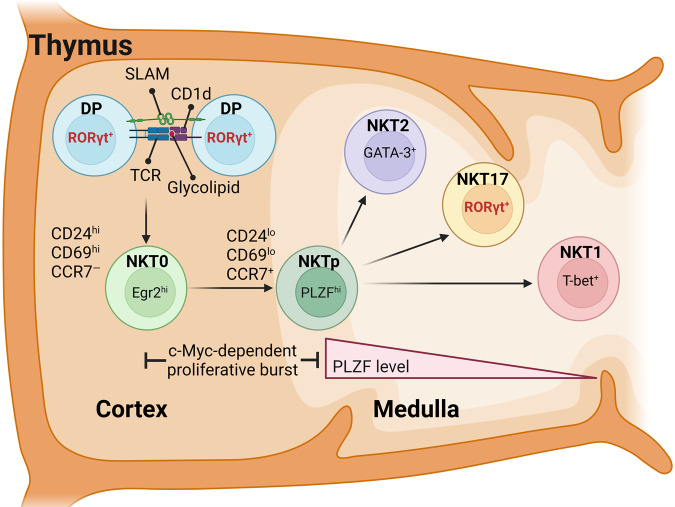
The development and differentiation of NKT17 cells in the thymus. In the thymic cortex, *i*NKT cells are positively selected by CD4^+^CD8^+^ double-positive (DP) thymocytes that present lipid antigens loaded on CD1d, a nonclassical MHC-I-like molecule. Selection into the *i*NKT cell lineage requires strong TCR signaling and homotypic interactions between signaling lymphocyte activation molecule (SLAM) family members. Post-selection *i*NKT cells are referred to as stage 0 or NKT0 cells, and they have high expression of Egr2, CD69, and CD24 but lack CCR7 expression. Immediately after positive selection, these progenitor cells undergo a c-Myc-dependent proliferative burst and proceed along a developmental pathway toward multipotent NKT cell precursors, designated as NKTp cells. These progenitor cells upregulate PLZF and CCR7 and downregulate CD24 expression before continuing terminal differentiation into three different *i*NKT subsets, namely, NKT1, NKT2, and NKT17 cells. NKT2 cells are GATA-3 positive and have the highest expression of PLZF, whereas NKT1 cells are T-bet positive and have the lowest PLZF expression. NKT17 cells are RORγt positive with intermediate PLZF expression.

The initial stages of *i*NKT cell generation parallel the development of conventional αβ T cells in the thymus. Both populations mostly arise from immature CD4^+^CD8^+^ double-positive (DP) thymocytes, at which stage they undergo positive selection^44–46^. Unlike that of conventional αβ T cells, however, the positive selection of *i*NKT cells is not mediated by thymic stromal cells in the cortex. Instead, *i*NKT cells are positively selected by glycolipid-loaded CD1d molecules that are expressed on thymocytes themselves and require homotypic costimulation by SLAM receptors (Fig. 1)^46,47^. Additionally, and unlike conventional αβ T cells that are generated by weak TCR engagement, *i*NKT cells are selected upon strong agonistic TCR signaling, which induces and requires the expression of the transcription factor early responsive gene 2 (Egr2)^48–50^. In agreement, the earliest *i*NKT-lineage cells in the thymus can be identified as Egr2^+^ cells that coexpress CD69 and bind to glycolipid-loaded CD1d tetramers^48–50^. These postselection immature *i*NKT cells are commonly referred to as stage 0 (ST0) cells or NKT0 cells^26,51,52^, and they express high levels of Nur77, indicative of the strong TCR signaling that mediates their generation **(**Fig. 1**)**^53,54^. Agonistic TCR signaling also highly upregulates the expression of PLZF, which is a critical nuclear factor required for the development and acquisition of effector function in *i*NKT cells^30,31^. ST0 *i*NKT cells express large amounts of CD24, a widely used marker for immature T cells^55^, but start downregulating CD24 expression upon further maturation that is associated with a c-Myc-dependent proliferative burst^56,57^. As a result of this expansion, three distinct subsets of *i*NKT cells, i.e., NKT1, NKT2, and NKT17, emerge; all of these subsets display a CD24^lo^ phenotype, but they differ in their effector function and expression of PLZF **(**Fig. 1)^27^. A recent study identified a previously unappreciated precursor population among CD24^lo^ *i*NKT cells, which is referred to as the NKT progenitor (NKTp) population and could give rise to all three *i*NKT subsets^58^. NKTp cells are marked by high-level expression of Egr2 and the chemokine receptors CCR7 and S1PR1 but lack effector function, even though they express large amounts of PLZF^59^. While the exact timing of *i*NKT subset specification remains unclear, the identification of the CD24^lo^ NKTp population suggested that it could occur during or immediately after positive selection-induced proliferation.

Because PLZF is induced upon TCR signaling, the distinct amounts of PLZF among different *i*NKT subsets have led to the assumption that NKT2 cells receive the strongest TCR signal during development because they have the highest level of PLZF (PLZF^hi^), whereas NKT1 cells are selected by the weakest TCR signal and have the lowest level of PLZF (PLZF^lo^) expression^31,32,60^. NKT17 cells, on the other hand, contain an intermediate amount of PLZF (PLZF^int^), and they are proposed to be selected by intermediate-strength TCR signals (Fig. 1)^31,32,60^. However, how the difference in TCR signaling strength can be generated when all *i*NKT subsets express the same canonical TCR genes, even though they differ in their CDR3 sequences^61^, has not been fully explained. Specifically, what defines an intermediate-strength signal so that some *i*NKT precursors do not diverge into either the NKT1 or NKT2 lineage but become directed toward the NKT17 lineage is unclear. Nonetheless, it is evident that the different levels of PLZF are associated with the expression of *i*NKT subset-specific transcription factors, such that PLZF^hi^ *i*NKT cells are GATA-3^hi^ and PLZF^int^ *i*NKT cells express RORγt, while PLZF^lo^ *i*NKT cells exclusively express T-bet (Fig. 1)^27,32,62^. Consequently, it has been considered a key issue to understand how TCR signals are translated into the induction of different transcription factors and, specifically in the context of NKT17 cell differentiation, how this induction would lead to the NKT17-specific expression of RORγt.

### NKT17 subset specification in the thymus

Among *i*NKT cells, RORγt is exclusively expressed in the NKT17 subset. Incidentally, RORγt is also highly expressed in DP thymocytes, which are the immediate precursors of immature *i*NKT cells^45,46,63,64^. In DP thymocytes, RORγt plays a critical role in cell survival by suppressing cellular metabolism^65^ and inducing antiapoptotic Bcl-xL expression^66,67^. Accordingly, RORγt-deficient mice fail to produce *i*NKT cells^45^ since their DP thymocytes cannot survive long enough to undergo successful rearrangement of the invariant Vα14-Jα18 chain that is encoded in the distal part of the TCRα gene^45,68^. In this context, the forced expression of transgenic Bcl-xL is sufficient to restore thymic *i*NKT cells, indicating that the primary role of RORγt in generating *i*NKT cells is to promote the survival of DP thymocytes^45^. Notably, such RORγt-deficient Bcl-xL-transgenic mice generate *i*NKT cells, but they still lack NKT17 cells. RORγt-deficient *i*NKT cells fail to become NKT17 cells because RORγt acts as the master regulator of NKT17 cell differentiation that equips these cells with a subset-specific phenotype and effector function^45^. Therefore, RORγt is expressed in both DP thymocytes and NKT17 cells but exerts dual functions in a cellular context-dependent manner.

Because RORγt is expressed in both DP thymocytes and NKT17 cells, it raises the question whether RORγt expression is ever turned off during the differentiation of DP thymocytes into NKT17 cells. Experimental data strongly support this hypothesis, and the current consensus is that positive selecting TCR signals terminate RORγt expression so that all *i*NKT subsets arise from a RORγt-negative precursor population. A recent study that employed a newly generated inducible Vα14-Jα18 TCR expression system reaffirmed this notion^69^. This genetically engineered mouse model permits monitoring of the development of a synchronized cohort of *i*NKT cells in vivo, and it revealed that positive selection of *i*NKT precursors terminates the expression of both the CD4 and CD8 coreceptors, rendering the cells CD4 and CD8 double-negative (CD4^–^CD8^–^), but also extinguishes their RORγt expression^69^. Thus, the immediate progeny of positively selected *i*NKT cells appear to be RORγt^–^CD4^–^CD8^–^ CD1d-tetramer-positive cells. These postselection immature *i*NKT cells then undergo induction of the transcription factor ThPOK and pass through a CD4^+^ stage that is common to all *i*NKT cells, after which they undergo lineage specification into different *i*NKT subsets^51,59,69^. Collectively, these results argue for dynamic regulation of RORγt expression during NKT17 cell differentiation, in which RORγt expression is transiently downregulated upon positive selection but then reinduced upon NKT17 lineage commitment.

**Fig. 2:**
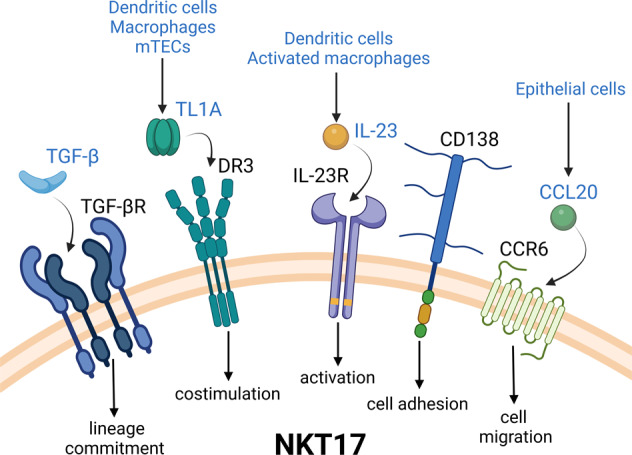
Surface molecules associated with the NKT17-specific phenotype and function. NKT17 cells can be identified by several cell-surface markers. CD138 (syndecan-1) is a heparan sulfate proteoglycan that is specifically expressed on NKT17 cells. CD138 interacts with extracellular matrix proteins, chemokines, cytokines, growth factors and integrins, among others, but its specific role, if any, in NKT17 cell biology remains unknown. CCR6 is a chemokine receptor associated with NKT17 cells that promotes their migration into tissues rich in its ligand CCL20, such as the skin, lungs, and lymph nodes. DR3 is a TNF receptor superfamily member that is expressed mostly by the NKT17 subset among thymic *i*NKT cells. Upon interaction with its endogenous ligand, TL1A, DR3 acts as a costimulatory molecule in context of the α-galactosylceramide-mediated activation of NKT17 cells. IL-23 receptor (IL-23R) is exclusively expressed on NKT17 cells, and its activation triggers IL-17A secretion. TGF-βR is a cytokine receptor for the multifunctional cytokine TGF-β. Analogous to its role in the Th17 cell polarization of CD4^+^ T cells, TGF-βR ligation augments NKT17 cell specification in *i*NKT cells. mTEC, medullary thymic epithelial cells.

Such a developmental trajectory of NKT17 cells necessitates a molecular explanation of how RORγt is re-expressed in NKT17 cells. Because of their IL-17-producing characteristics, NKT17 cells have frequently been compared to IL-17-producing CD4^+^ Th17 cells^39,70^. Th17 cells can be generated from naive CD4 T cells by TCR activation in the presence of the cytokines TGF-β and IL-6^71^. Thus, it seems feasible that the same cytokine combination would also drive RORγt induction and NKT17 cell differentiation. This presumption, however, is not quite correct. While NKT17 cells express high levels of TGF-β receptors and show markedly elevated phosphorylation of SMAD2/3 when freshly extracted from the thymus^69,72^, IL-6 is not required for the generation of NKT17 cells^72–74^ (Fig. 2). Moreover, in humans, in which NKT17 cells were first described as CD161^+^ *i*NKT cells, the differentiation and acquisition of effector function of NKT17 cells depend not only on TGF-β but also on IL-1β and IL-23^75^. Importantly, the IL-23 receptor is highly and selectively expressed on NKT17 cells, so it has been considered a prominent marker for the NKT17 subset of *i*NKT cells^73,76^, whereby IL-23 receptor signaling possibly plays a critical role in NKT17 lineage commitment rather than maintenance^77^. Overall, TGF-β is a common denominator for both mouse and human NKT17 cells as well as a common denominator between Th17 and NKT17 cells, but there are variations in the requirements for costimulatory cytokines between these cells. In this regard, it remains unclear how the coordinated effects of TGF-β and other cytokines contribute to the induction of RORγt and the specification of the NKT17 subset. In TGF-β signaling, for example, conditional deletion of SMAD4 but not TRIM33 was shown to impair NKT17 cell generation, although the downstream target genes of SMAD activation have yet to be identified^72^. The same study showed that constitutive expression of TGF-β promoted the survival and accumulation of NKT17 cells in peripheral lymph nodes but did not affect NKT17 cell generation in the thymus^72^. Thus, the cytokine requirements for NKT17 cells can differ depending on the tissue environment, and the downstream targets of NKT17-specifying cytokines remain to be determined.

### Nuclear factors controlling NKT17 subset specification

The distinct TGF-β signature in NKT17 cells suggests that cytokine signaling contributes to *i*NKT subset specification, either in addition to or in concert with TCR signaling^69,72^. In fact, it is likely that positive selection and lineage specification of *i*NKT cells are disparate events, as is the case for the thymic generation of conventional CD4 and CD8αβ T cells^78^. In this setting, TCR signals would be mostly required for positive selection, and cytokine signals would determine the subset identity, as recently proposed^69^. However, it is unclear how only some *i*NKT cells would respond to a specific cytokine, such as TGF-β, to commit to a particular subset, while other *i*NKT cells are nonresponsive to the same cytokine. Whether the strength and/or kinetics of the preceding or concomitant TCR signal play a role in this process is a possibility that can be tested. However, a clear distinction between or overlap of the cytokine versus TCR contributions in *i*NKT subset differentiation has yet to be established. As such, assessing how the downstream signaling pathways of TCR and cytokine signaling intersect to establish *i*NKT subset identity remains an area of active research.

NKT17 cells express intermediate levels of the transcription factors PLZF and Egr2, whose abundances correlate with the strength of TCR signaling^32,50,60^. Hypothetically, intermediate-strength TCR signals could be necessary to transition immature *i*NKT cells into a TGF-β signaling-permissive state so that they can induce RORγt in response to TGF-β. Weaker or stronger TCR signals, on the other hand, would keep immature *i*NKT cells refractory to TGF-β and thus prevent the induction of RORγt. Such a scenario implies that RORγt expression would be actively suppressed in non-NKT17 lineage-committed *i*NKT cells and that either PLZF or Egr2 could be involved in this process. However, both PLZF- and Egr2-deficient mice fail to generate mature *i*NKT cells^30,31,49,50^, making it difficult to test the roles of these transcription factors in *i*NKT subset specification.

Another prominent transcription factor whose expression is associated with TCR signal strength is the zinc finger transcription factor ThPOK^79^. ThPOK is absent in immature thymocytes but highly upregulated upon strong and persistent TCR signaling^80,81^. In agreement, CD4-lineage T cells, which require strong/persistent TCR signaling for their generation, express high levels of ThPOK, and their differentiation depends on ThPOK^80,82^. Notably, ThPOK is also highly expressed in *i*NKT cells, which is consistent with their requirement for strong agonistic TCR signaling^53,83,84^. However, unlike conventional CD4 T cells, which are virtually absent in ThPOK-deficient mice^80,82,85^, *i*NKT cells still develop in the absence of ThPOK, indicating that ThPOK presumably plays distinct roles in *i*NKT cells versus conventional CD4 T cells^83,86^. In this regard, ThPOK suppresses cytokine receptor signaling by controlling the expression of SOCS family molecules^87^, so ThPOK can bridge TCR signaling with cytokine signaling in *i*NKT cell differentiation. The *i*NKT subset analysis of ThPOK-deficient *i*NKT cells bolstered this possibility, noting that mice with a missense mutation in ThPOK or germline deficiency in ThPOK displayed dramatic increases in the frequency and number of thymic NKT17 cells^83,88,89^. ThPOK deficiency was also shown to promote NKT17 cell differentiation in the spleen and liver compared to control expression^88,89^. Conversely, the forced expression of ThPOK was found to potently suppress RORγt expression and the differentiation of IL-17-producing *i*NKT cells^89^. Thus, ThPOK acts as a suppressor of RORγt expression during *i*NKT subset specification, revealing a new layer of control in NKT17 cell generation. Whether ThPOK suppresses RORγt expression directly by interfering with its transcription or indirectly by modulating cytokine responsiveness or the expression of other factors is an important question that needs to be addressed.

The runt-family transcription factor Runx3 is a major target of ThPOK^90^. Because ThPOK suppresses Runx3 expression^87,90,91^, under normal circumstances, the expression of Runx3 and ThPOK is mutually exclusive^92^. As a corollary, Runx3 is mostly absent when ThPOK is expressed, as is the case in both CD4 cells and *i*NKT cells^85,93^. Interestingly, ThPOK-deficient *i*NKT cells show marked induction of Runx3 that is associated with ectopic CD8 coreceptor expression and altered effector function^93^. In this regard, it could be either the absence of ThPOK or the increased expression of Runx3 that promotes RORγt expression and NKT17 differentiation in ThPOK-deficient mice. Further experimental data and new mouse models, such as *i*NKT-specific overexpression of Runx3, are necessary to discriminate these possibilities. Nonetheless, a requirement for Runx3 in *i*NKT cell generation could be excluded based on the observation that Runx3-conditional knockout mice that lack Runx3 are mostly unaffected in their thymic *i*NKT cell differentiation^93^. Thus, unlike ThPOK, Runx3 is not a major contributor to the differentiation of *i*NKT cells.

In addition to Runx3, the Runx family members include Runx1 and Runx2^94^. However, only Runx1 and Runx3 are expressed in lymphocytes^85,93,95^. In contrast to Runx3, which is primarily expressed in CD8 T cells and NK cells, Runx1 is highly expressed in CD4 T cells and *i*NKT cells^93,96^, suggesting that Runx1 rather than Runx3 could play a role in *i*NKT cells. Indeed, Runx1 deficiency in preselection thymocytes results in a complete block of *i*NKT cell development at the immature DP stage^45^, while conditional deletion of Runx1 with PLZF-Cre severely impairs the functional maturation of positively selected *i*NKT cells^97^. These findings support Runx1 as a nonredundant requirement in *i*NKT cell generation and differentiation. Since RORγt is suppressed by ThPOK, which in turn is antagonized by Runx3, Runx1 could potentially interfere with RORγt expression. In fact, Runx1-mediated activation of RORγt was previously demonstrated in IL-17-producing Th17 CD4 T cells^98^, and it is reasonable to assume that a similar pathway also operates in *i*NKT cells. In agreement, analyses of Runx1-deficient mice have shown that their *i*NKT cells exhibited significant reductions in the frequency and number of NKT17 cells and that cytokine production was selectively impaired in the NKT17 subset but not in NKT1 or NKT2 cells. Collectively, these results strongly suggest that Runx1 is a positive regulator of the transcriptional program that governs NKT17 cells.

Mechanistically, however, Runx1 could be controlling not only the expression of RORγt but also that of other genes associated with NKT17 cell differentiation. In this regard, it was interesting to find that the expression of c-Maf, a transcription factor that is highly enriched in NKT17 cells^99^, was markedly reduced in Runx1-deficient *i*NKT cells. Runx1 deficiency also induced a significant decrease in the expression of BATF, a transcription factor that promotes the in vitro differentiation of CD4^+^ Th17 cells^100^, resulting in a dramatic loss of IL-17 production in NKT17 cells. Reciprocally, BATF overexpression has been shown to skew the *i*NKT subset composition toward NKT17 cells, further unraveling the complex regulatory pathway of NKT17 cell differentiation that is controlled by Runx1 and associated transcription factors. Evidently, there is an ever-growing body of regulatory factors identified to be involved in the subset differentiation of NKT17 cells, and they include the transcriptional repressor NKAP^101^, which is specifically required for the differentiation of NKT17 but not NKT1 or NKT2 cells, as illustrated by the marked paucity of NKT17 cells in NKAP-deficient mice^101^. The mRNA-binding protein Roquin^102^ and the transcription factor Bcl11b^103^, on the other hand, are negative regulators of NKT17 cell generation, that could play yet to be assessed roles in RORγt expression at the posttranscriptional or transcriptional level. Connecting these factors into a comprehensive network is a daunting task, which will require comprehensive pathway analyses together with in-depth bioinformatic approaches.

### Surface markers that identify NKT17 cells

The term NKT17 cells was coined in the seminal study by Michel and colleagues^104^, in which IL-17-producing *i*NKT cells were identified in the spleen, liver, and lungs as NK1.1-negative *i*NKT cells. Thus, starting early on, the phenotype of *i*NKT cells, such as expressing the surface marker NK1.1 expression, has been closely associated with effector function. However, not all NK1.1-negative *i*NKT cells are NKT17 cells. In fact, the NK1.1-negative *i*NKT population contains two distinct subsets that can be distinguished based on CD44 expression^26,70^. Because CD44 expression is thought to be an acquired trait upon thymic maturation, CD44^–^NK1.1^–^ thymic *i*NKT cells are commonly referred to as stage 1 (ST1), while CD44^+^NK1.1^–^ thymic *i*NKT cells correspond to stage 2 (ST2). *i*NKT cells that express both CD44 and NK1.1 (CD44^+^NK1.1^+^) are considered to have undergone terminal differentiation, and they are known as stage 3 (ST3) *i*NKT cells^9,26,70^. Detailed analyses of their functional characteristics place IFNγ-producing NKT1 cells into the ST3 compartment. In contrast, NKT17 cells are excluded from the ST3 subset, and they correspond mostly to ST2 cells. However, not all ST2 cells are NKT17 cells, as this population is heavily contaminated with NKT2 cells^105^. Therefore, alternative or additional markers are required to identify NKT17 cells.

Based on CD4 coreceptor expression, *i*NKT cells can be either CD4-positive or CD4-negative^106^. IL-17-producing *i*NKT cells are mostly found in the CD4-negative compartment, so NKT17 cells have been proposed to be phenotypically CD4^–^NK1.1^–^ cells^76^. In fact, the lack of both CD4 and NK1.1 expression has been employed for a long time as a surrogate marker for NKT17 cells^48,76,107^. Alternatively, the IL-2/IL-15 receptor β-chain, i.e., CD122, is exclusively expressed on NKT1 cells, and combined with the observation that all NKT2 cells express CD4, the use of CD122 and CD4 can discriminate the three major subsets of *i*NKT cells. Accordingly, NKT1 cells are CD122^+^ and NKT2 cells are CD4^+^CD122^–^, while NKT17 cells are identified as CD122^–^CD4^–^ double-negative (DN) cells^27,48^. Accordingly, the CD122^–^CD4^–^ DN compartment corresponds mostly to RORγt^+^ *i*NKT cells^108^.

However, further in-depth studies of IL-17 production have revealed that a significant fraction of NKT17 cells are also found among CD4^+^ *i*NKT cells. Such CD4-expressing NKT17 cells have been reported in the thymus and lymph nodes of both BALB/c mice and C57BL/6 mice^29^, and ~10% of NKT17 cells residing in the mesenteric lymph nodes are CD4-positive^72^. The relative frequency of CD4-positive NKT17 cells among total NKT17 cells in different tissues is still debated, but a lack of CD4 expression alone cannot be employed to identify all NKT17 cells^29^. Consequently, alternative approaches making use of markers other than CD4 to identify NKT17 cells have been reported.

A powerful tool for NKT17 cell identification was devised using the differential expression of ICOS (CD278) and the activation-associated glycoform of CD43 (CD43HG) in *i*NKT cells^29^. Accordingly, NKT1 cells are identified as CD43^–^ ICOS-low cells, and NKT2 cells are identified as CD43-intermediate ICOS^+^ cells, whereas NKT17 cells are CD43^+^ ICOS-high cells^29^. To confirm correct identification, *i*NKT subsets stratified by ICOS versus CD43HG staining were assessed for intracellular T-bet and RORγt staining and found to match their expected subset characteristics^29^. Along these lines, NKT2 cells that were purified based on CD43 and ICOS expression produced negligible levels of IL-17A, affirming that these markers accurately identified and excluded specific subsets^29^. Recently, a different set of surface markers was employed to identify NKT17 cells in FVB/N mice; NKT1 cells were first excluded from total *i*NKT cells based on their lack of PD-1 and ICOS expression and then gated on CD4-negative but CD27^+^ cells among the remaining PD-1^+^ ICOS^+^ *i*NKT cells^38^. Thus, using a combination of markers and gating strategies permits correct discrimination of individual *i*NKT subsets. Such an approach has turned out to be highly effective, but it is also cumbersome and complicated because multiple markers are required to identify the desired *i*NKT subset.

Accordingly, it would be more effective if the target *i*NKT subset could be identified by a single marker that is exclusively expressed by that particular *i*NKT cell population. CD122 corresponds to such a marker for NKT1 cells because it is expressed by only this specific *i*NKT subset and is incidentally also required for IL-15 signaling, which induces NKT1-specific T-bet expression^109,110^. In this regard, surface markers that are related to the function or a developmental requirement of a given *i*NKT subset could serve as faithful markers to identify that specific subset among other *i*NKT cells.

*i*NKT subsets are distinct in their tissue tropism and residency, given their differential chemokine receptor and cell adhesion molecule expression^10,41,43^. As such, NKT17 cells are especially enriched in the skin and lungs^27,43,104^. The chemokine receptor CCR6 is a major chemokine receptor in Th17 cells^111^ and NKT17 cells^107^, which could explain the enrichment of these subsets in the skin epithelium and mucosal tissues in which CCL20, the ligand for CCR6, is highly expressed (Fig. 2)^112^. In agreement, CCR6 mRNA transcripts were found exclusively in NKT17 cells among different *i*NKT subsets^107^. However, surface CCR6 expression is heterogeneous among NKT17 cells^72^, indicating that CCR6 can mark NKT17 cells but that not all NKT17 cells are necessarily CCR6 positive^72^. Such heterogeneity in CCR6 expression renders this chemokine receptor an unpredictable marker for NKT17 cells. Likewise, neuropilin-1, CD103, and CD121a have been proposed as promising candidates to identify NKT17 cells^72^. However, all of them were later found to be either heterogeneously expressed among NKT17 cells or not entirely specific to the NKT17 subset. Thus, a marker that could identify NKT17 cells has not been successfully identified among cytokine and chemokine receptors or cell adhesion molecules.

Serendipitously, CD138 (Syndecan-1) was recently discovered to be an NKT17-specific surface marker (Fig. 2)^113^. In support of this notion, only CD138^+^ *i*NKT cells produce IL-17 among all *i*NKT cells^113^. CD138 is a transmembrane heparan sulfate proteoglycan that is highly expressed on epithelial cells and plasma cells^114^. In T cells and thymocytes, CD138 is expressed on only a subset of mature CD4, CD8 DN thymocytes, among which the majority of CD138^+^ cells were found to be NKT17 cells, with a minor population of γδ T cells^108^. Currently, the role of CD138 in NKT17 cells is not fully understood. CD138 usually interacts with cell matrix proteins, cytokines, and growth factors^114^, so it could play regulatory roles in the tissue distribution, proliferation, or activation of NKT17 cells. However, CD138-deficient *Sdc1*^–/–^ mice do not show any defects in the generation of NKT17 cells, and IL-17 production by *Sdc1*^–/–^ NKT17 cells is also unimpaired^108,113^. Rather, both the frequency and number of thymic NKT17 cells are modestly increased, suggesting that CD138 does not play a major role in NKT17 cells and that even if it did, it would play a negative regulatory role^108^. To this extent, CD138 expression is mostly considered as a surface marker for NKT17 cells, with no clear biological function in NKT17 cells yet elucidated.

The ongoing search for a functional marker recently yielded the surprising discovery of Death Receptor-3 (DR3, also APO-3) as an NKT17 subset-specific molecule^115^ (Fig. 2). DR3 is a member of the TNF receptor superfamily that is activated by its only known ligand, TL1A, to trigger proinflammatory and apoptotic downstream signaling^116^. TL1A is primarily produced by antigen-presenting cells, such as dendritic cells and macrophages, but also by thymic medullary epithelial cells^116^, suggesting a potential role for the DR3-TL1A signaling pathway in T cell development (Fig. 2). Along these lines, DR3 was previously reported to be highly expressed on Foxp3^+^ CD4^+^ T regulatory cells (Tregs) to promote their expansion and partly contribute to their effector function^117^. As such, DR3 ligation with agonistic antibodies was shown to result in a dramatic expansion of Foxp3^+^ Treg cells in vivo^117^ and to ameliorate disease severity in acute GVHD settings^117^. A mouse model of constitutive TL1A expression also exhibited expansion of the pool of DR3-expressing cells, identifying group 2 innate lymphoid cells (ILC2s) as a target of TL1A^118^. Here, the excessive production of TL1A resulted in a significant increase in the ILC2 population, concomitant with an IL-13-mediated allergic immune response^118^. The expression of DR3 on thymic *i*NKT cells, specifically on NKT17 cells, however, had not been documented until recently^115^. Notably, NKT17-specific DR3 expression was mostly limited to NKT17 cells in the thymus, as DR3 expression is rather promiscuous on *i*NKT cells in peripheral tissues. While DR3 remains highly expressed on NKT17 cells, there is a substantial amount of DR3 expression on *i*NKT subsets in the lymph nodes and lungs, among other tissues^115^. However, what drives DR3 expression on peripheral *i*NKT cells that lack RORγt remains to be determined.

Because of the subset-specific expression of some cytokine receptors, such as CD122 for NKT1 cells and IL-17RB for NKT2 and NKT17 cells^119,120^, it is not surprising that there would be a proprietary cytokine receptor for NKT17 cells, which turned out to be DR3^115^. The molecular basis of NKT17-specific DR3 expression could be traced back to RORγt, which was sufficient to induce DR3 expression on conventional αβ T cells and non-NKT17 cells in thymocytes of RORγt-transgenic mice^115^. These results suggest that DR3 expression is a direct target of RORγt. In agreement, it was previously reported that DR3 is specifically expressed on Th17 cells, the only T helper cell subset that expresses RORγt^121^. Regarding the requirement for and effect of DR3 in NKT17 cells, however, we are only beginning to understand the role of DR3. While in vitro stimulation with agonistic anti-DR3 antibodies is sufficient to induce the early activation marker CD69, DR3 ligation alone fails to induce a sufficiently strong response to boost IL-17 production. Instead, DR3 acts more like a costimulatory molecule, as it was found to dramatically amplify the effect of α-GalCer stimulation and to increase IL-17 production and cell proliferation of thymic NKT17 cells^115^. Therefore, DR3 represents a new class of costimulatory molecules on thymic NKT17 cells that can serve as both a marker and a trigger of a specific *i*NKT subset.

### Perspectives

*i*NKT cells undergo terminal differentiation in the thymus, upon which they egress into peripheral organs to establish tissue residency^9,10,43,59^. Generally, the subset identity of *i*NKT cells is considered developmentally fixed, so that NKT17 cells do not further differentiate into NKT1 cells and NKT2 cells or vice versa in the periphery^27^. However, the *i*NKT subset composition in the thymus is strikingly different from that in peripheral organs, and it also varies among different organs^10^. In this regard, NKT1 cells are highly enriched in the liver, while NKT17 cells accumulate in the lymph nodes and lungs^43^. Several distinct but not mutually exclusive models have been proposed to explain the tissue-specific distributions of individual *i*NKT subsets. A straightforward explanation would be that the thymic export and tissue tropism of *i*NKT cells differ among subsets. For example, some *i*NKT cells could efficiently exit the thymus and migrate to their target tissues, while others would be impaired in thymic egress and become thymus resident. In this regard, NKT2 cells express large amounts of CD69, which retains them in the thymus^33^, whereas NKT1 and NKT17 cells express the chemokine receptors CXCR3 and CCR6, respectively, which could attract them into peripheral tissues where their ligands are highly expressed^48^. CXCR6 is a chemokine receptor that is important for the survival and maintenance of *i*NKT cells in the liver, whereas the factors required for *i*NKT cell homing to the lungs have yet to be determined^10,41^. Thus, differences in thymic egress and tissue tropism could cause the differences in the *i*NKT subset composition between the thymus and peripheral organs.

Another attractive hypothesis is that each tissue environment provides unique survival signals that are tailored to each *i*NKT subset, resulting in the preferential survival and accumulation of a particular subset. In this regard, NKT17 cells reportedly prefer IL-7 over IL-15 signaling for their survival^42,110,122^, and this IL-7 dependence would cause enrichment in the lymph nodes, where IL-7 is abundantly expressed^123^. Why NKT17 cells would be more responsive to and dependent on IL-7 than the other *i*NKT subsets is not fully understood. A recent study demonstrated that forkhead box protein O1 (FoxO1) plays a critical role in NKT1 and NKT2 cells but not in NKT17 cells^124^. Notably, expression of the IL-7 receptor, which is highly abundant on all *i*NKT cells, is driven by FoxO1 in NKT1 and NKT2 cells but not in NKT17 cells. Instead, it turned out that it is RORγt which promotes IL-7 receptor expression on NKT17 cells^124^.

Overall, RORγt remains a critical master regulator of NKT17 cells that guides not only the generation and differentiation but also the survival and homeostasis of this *i*NKT subset. The observation that RORγt also controls the expression of surface molecules that mark NKT17 cells provides further evidence that the phenotype and function of NKT17 cells are closely associated with each other. Considering that the role of NKT17 cells is still being unraveled, it is important to decipher which NKT17 markers are functional and contribute to NKT17 biology, and which molecules are passenger markers with no apparent function. Further synthesis of this information will provide us with a better understanding of NKT17 cells in immunity.
